# Navigation in darkness: How the marine midge (*Pontomyia oceana*) locates hard substrates above the water level to lay eggs

**DOI:** 10.1371/journal.pone.0246060

**Published:** 2021-01-25

**Authors:** Chun-Gin Chang, Chia-Hsuan Hsu, Keryea Soong

**Affiliations:** Department of Oceanography, National Sun Yat-sen University, Kaohsiung, Taiwan; Kaohsiung Medical University, TAIWAN

## Abstract

Finding suitable habitats for specific functions such as breeding provides examples of key biotic adaptation. The adult marine midge *Pontomyia oceana* requires an extremely specific habitat, i.e., hard substrates above water in shallow water, to deposit fertilized eggs. We investigated how these sea surface-skimming insects accomplished this with a stringent time constraint of 1–2 h of the adult life span in the evenings. We observed that in artificial containers, midges aggregated at bright spots only if the light was not in the direction of the sea. This behavior could potentially attract midges toward the shore and away from the open water. Experiments were performed in the intertidal zone in southern Taiwan to test three hypotheses explaining such behavior: gradients of temperature and CO_2_, and soundscape. No differences were observed in moving directions or aggregation of midges under artificial temperature and CO_2_ gradients. However, midges preferred sounds at 75 Hz compared with other frequencies (all ≤300 Hz) as observed in a field experiment involving floating traps with loudspeakers. Moreover, when background noise was experimentally masked using white noise of all frequencies, midges were significantly more likely to aggregate at bright spots in the direction of the sea than in the absence of white noise. These results establish that sound is used by midges to navigate in dark seas and move toward the shore where exposed hard substrates are in abundance. Marine mammals present well-known cases of sound pollution at sea; here the finding in the insignificant marine midge is just the harbinger of the potential effects noise at shore may have to affect critical reproductive stages of marine organisms.

## Introduction

Understanding how organisms, especially marine animals, return to natal habitats to reproduce, is often a fascinating challenge [1, 2]. Studies have determined that in groups where adults are sessile or less mobile, and disperse by planktonic larvae, suitable environmental cues the metamorphosis to benthic life stages [3, 4]. More mobile organisms may have other mechanisms because they have more control of their locomotion and can move toward a certain direction. Various traits of migratory organisms, such as sea turtles and salmon, have been discovered that help them to travel across long distances [5, 6]; complicated mechanisms are often involved. For shorter distances, organisms must choose the correct direction, such as in the case of gravid land crabs approaching the sea and returning to original habitats after releasing larvae [7–9]. Similar situation applies to sea turtle hatchlings heading from their beach breeding grounds to the sea [10]. Because of the short distance involved, the mechanism may be simple, especially in comparison with that used by long-distance travelers. Nevertheless, few studies have investigated this small-scale spatial movement in temporally short durations [11]. The short duration of this behavior by itself is often a challenge to researchers.

The marine midge *Pontomyia oceana*, living in shallow reefs, emerges into the adult stage a few hours after sunset following a semilunar rhythm, after spending most of its life as a benthic larva living in tubes made of debris [12]. During their short adult stage, which lasts approximately 1–2 h, they eclose, find mates, and copulate on the water surface. Male midges then drag immobile female midges to a hard substrate to lay eggs when the tide was low. The male midges then return to locate more females [12]. We aimed to investigate how male midges together with their dragged females find hard substrates in darkness.

In southern Taiwan, marine midges are found in a wave-protected intertidal zone, where abundant exposed substrates exist in most directions at low tide. The potential of strong selection to locate a suitable substrate was first discovered at a relatively unprotected coast stretch of remote Dongsha Island (South China Sea). There, newly emerged midges moving in the wrong direction in the shallow sea would encounter the expanse of the South China Sea, that is, they do not have a chance to reproduce successfully if they fail to find a suitable substrate within a short period of time. How they navigate in the right direction in the last and critical hours of their lives remains unclear. Were there any evolutionary traits to find suitable breeding habitats that could increase their reproductive fitness?

The intertidal and shallow waters comprise numerous environmental gradients, which organisms could use to navigate. However, underwater characteristics can rarely be used by the surface-skimming adult *P*. *oceana*. In the case of small organisms, especially those with extremely limited adult lifespans, the number of potentially suitable environmental cues are few. The time of the evening emergence of *P*. *oceana* is known to be controlled by an endogenous circasemilunar rhythm [13, 14]. Thus, the mechanism of orientation and navigation must work during both the full moon and new moon. Despite all constraints, some environmental factors are still exploitable for navigation toward hard substrates above the water surface.

Temperature can be a valuable determinant because land and water dissipate heat at different rates, and temperatures differ between these realms. Temperature differences of land and water can be remotely detected by organisms as thermal radiation of the electromagnetic spectrum [15].

The CO_2_ concentration in the atmosphere is another possible locomotory cue, because a gradient is supposed to exist along the land–sea axis due to the photosynthesis activity of plants on land [16]. Sound is another possibility because waves breaking at shores and motion of the water at shallow depths usually produce a characteristic sound [17].

From a physiological perspective, temperature, CO_2_, and sound are all known to be detectable by at least some dipteran insects where marine midges belong to. Insects use environmental signals to locate prey and mates (temperature: [18]; CO_2_: [18–20]; sound: [21, 22]). One or more of these factors may be used by *P*. *oceana* to infer the location of suitable habitats in the darkness of the sea. We tested these environmental factors in this study to determine which of them is used by *P*. *oceana*.

## Materials and methods

### Midge collection

Marine midges used in this experiment were collected by light trap in shallow water at Wanliton (21°59’45”N, 120°42’19”E), south Taiwan, during the evenings bracketing new moon and full moon. A collection permit was obtained from Kenting National Park Headquarters.

Light near the water surface can attract males, and immobile females can copulate with them. Because of their short adult life span, we performed experiments immediately after they were collected.

### Light experiment

**Fig 1:**
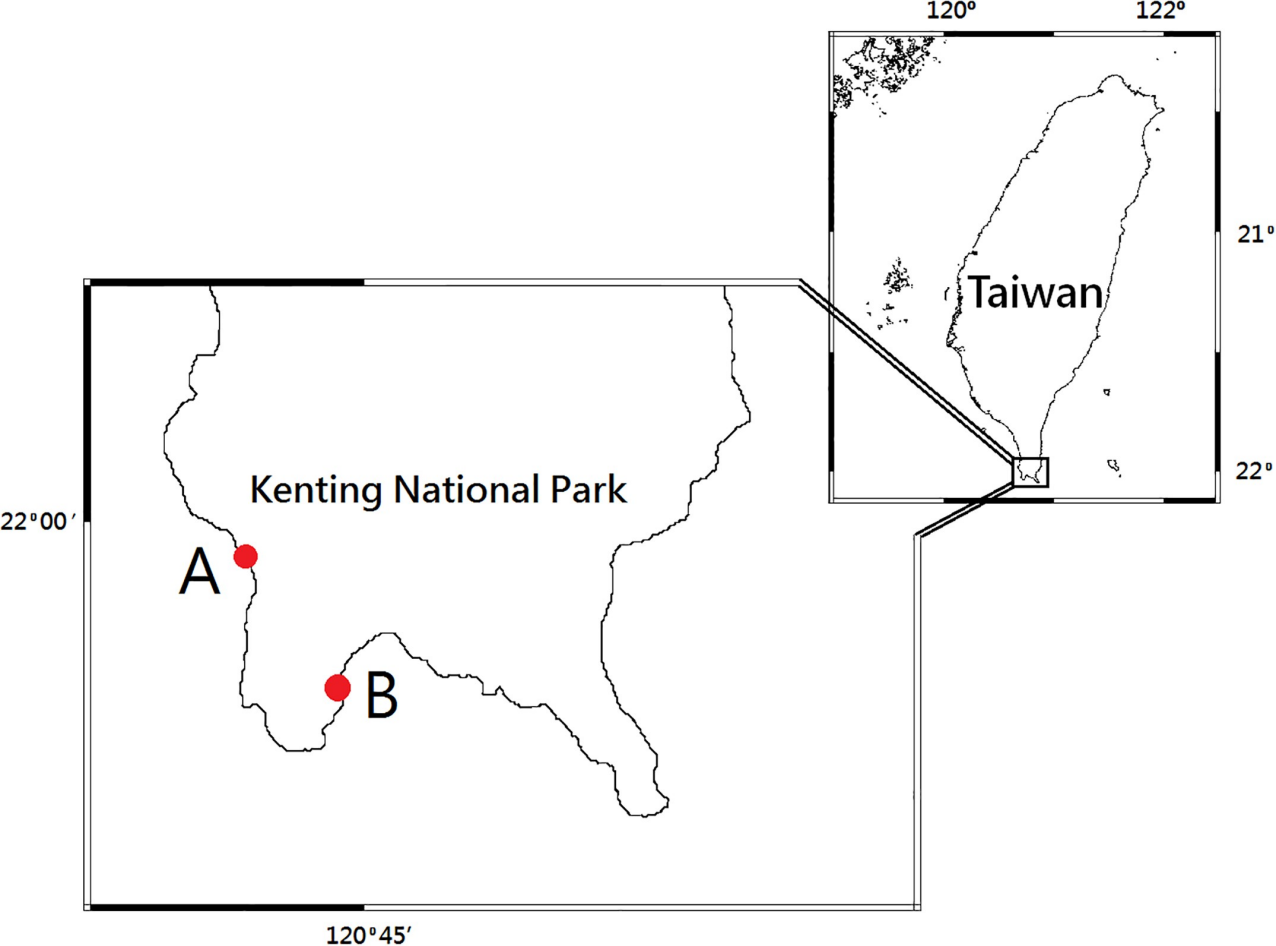
Study sites. (A) Wanliton (B) Hobihu.

For the light experiment, we used three treatments, namely light from the direction of the sea; light from directions at 90°, 180°, and 270°; and no light. The actual direction of the sea was dependent on the location of the experiment. The locations used in this experiment were where the direction of the sea was either to the west (Wanliton, 21°59’45”N, 120°42’19”E), south, or east (Hobihu, 21°56’26”N, 120°44’42”E) (Fig 1). They were approximately 8 km apart, reachable in approximately 15 min by car.

A round basin, 60 cm in diameter and 20 cm in height, was used in this experiment to examine the aggregation of midges under various conditions. A piece of black cloth was used to block ambient light. A sheet of sticky insect paper was fixed on the inner perimeter of the basin near the water line by using double-sided sticky tapes so that midges coming into contact with it adhered to the surface. A hole in the center of the black cloth allowed a funnel to be inserted so that live midges could be introduced into the center of the basin without compromising the darkness of the container. A layer of seawater in the container allowed midges to move freely on the surface. Two minutes after introducing midges, when all midges adhered to the insect paper, the experiment was terminated. The paper was then removed and marked, and the number of male and female midges on each of the 18 sections, each representing 20°, was counted. Additional tests under similar or different conditions were performed until midges became inactive later in the evening.

A single 5-mm white LED light was used on the perimeter of the containers to attract marine midges in the basin; it had an intensity of 27.8 lux (0.5 μmol/m^2^/s) at a distance of 5 cm, respectively. The light was arranged either in the direction of the sea or other directions.

### Temperature experiment

In preliminary observations using thermal images, the exposed reef substrate above the water surface was observed to have a temperature lower than that of sea water by several degrees at the time when midges emerge; that is, a few hours after dark in the end of October 2013. Therefore, our experimental set up compared ambient- and lower-temperature regions in the experimental basin.

In the temperature experiment, low temperatures were generated locally by using cool pads (3M Nexcare First Aid). They created a cool region of approximately 8 cm in length on the side of the basin. The temperature actually reached was 22.4°C–22.8°C, in comparison with 25.6°C–25.8°C in other parts of the tanks. The setup of the basin was identical to that in the light experiment, except the treatment was temperature, and no light spots were used.

### CO_2_ experiment

**Fig 2:**
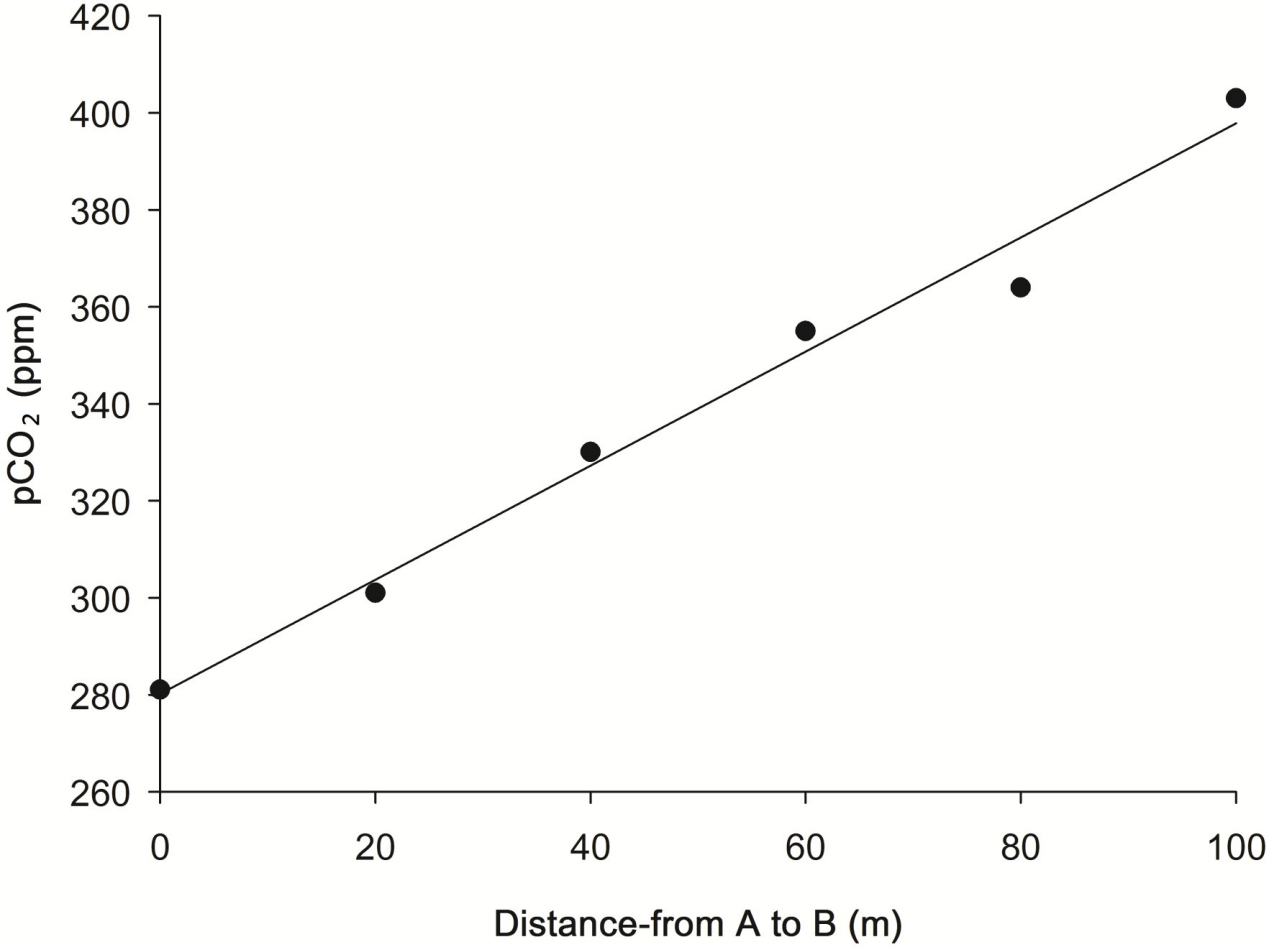
pCO_2_ gradient in a transect on the reef flat perpendicular to the coastline at Wanliton. A: sea end, B: coast end.

In a preliminary observation, a 122-ppm difference in CO_2_ in the air was observed along a line transecting perpendicular to the coast from approximately 1-m deep on the reef (281 ppm) toward the coast for 120 m (403 ppm, above sea level), at Wanliton, southern Taiwan (Fig 2). pCO_2_ was measured using Q-T_RAK_ Model 7545 by TSI Incorporated (Shoreview, Minnesota State, USA).

In the experiment, artificial CO_2_ gradients were generated by pumping air through a bottle with oversaturated Ca(OH)_2_ to absorb CO_2_ and by a control group with normal air supply without such an absorption bottle. These two sources of air were simultaneously injected into the two ends of a 4-m-long, 10-cm-diameter PVC pipes. The center of the pipe had a hole to let the air out, and the horizontal pipe was designed to be half-filled with seawater for marine midges to move on the water surface. The actual CO_2_ concentration was 365 ppm at the control end and 195 ppm at the experimental end. A black cloth was used to cover the openings of the pipe so that light did not affect the movement of midges. Midges moving to the two ends of the pipe were collected in two respective cups. CO_2_ was lower either at the end facing the land or the sea for 15 replications each. A preliminary test where both ends had normal air was run 10 times. A nonparametric Wilcoxon signed rank test was used to compare the data of this experiment.

### Sound experiment

**Fig 3:**
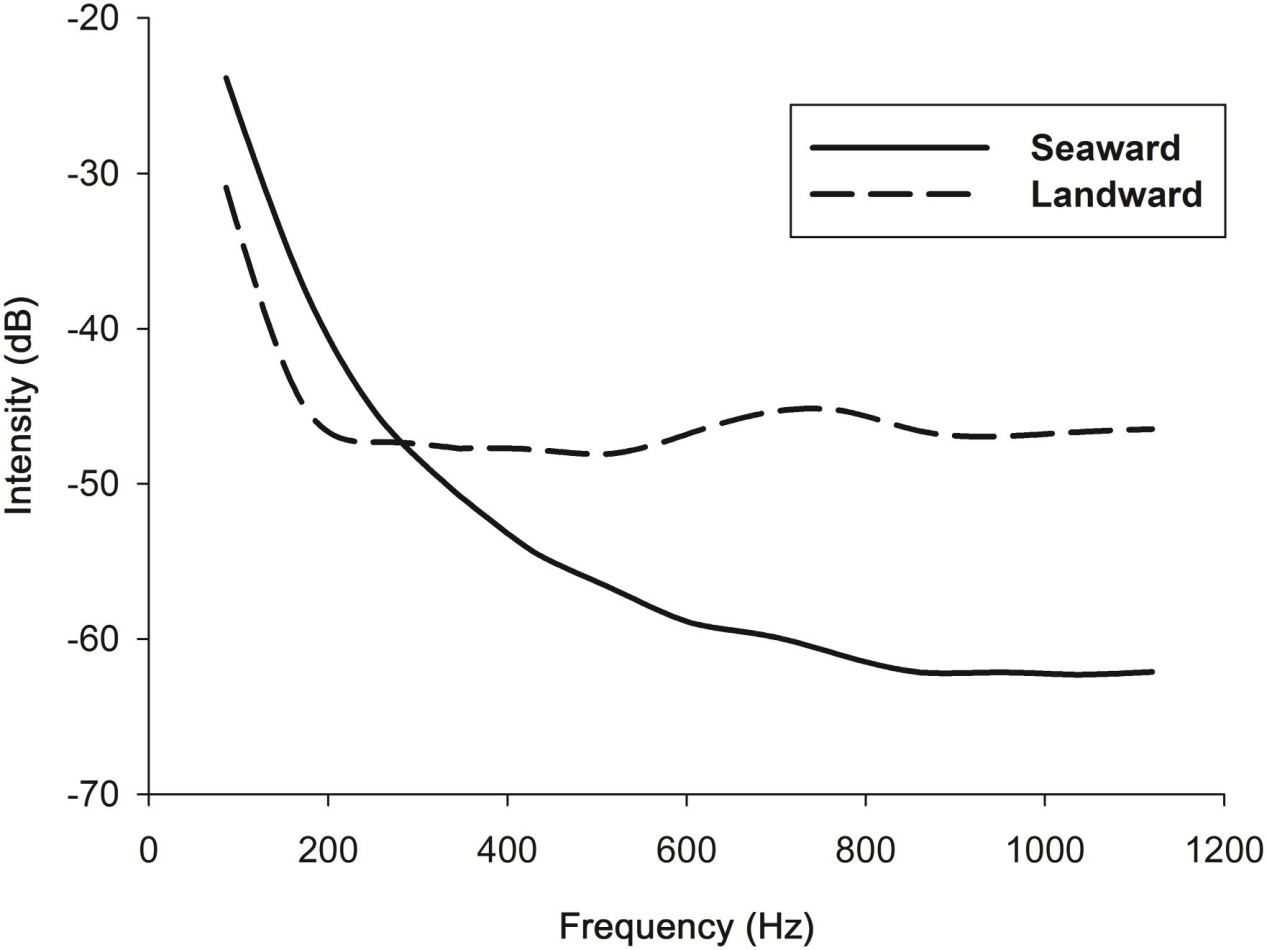
Sound intensity detected by receivers pointing seaward versus landward at the shore of Wanliton, south Taiwan.

In a preliminary survey, the background noise was measured to be in the range of 40–65 dB. Using a directional sound receiver, we observed that measurements pointing toward the sea had higher dB levels than those pointing toward the land between 0 Hz and 280 Hz. However, the trend was reversed at >344 Hz, after averaging five recordings at the coast of Wanliton (Fig 3).

In the first part of the experiment, floating traps with portable speakers (Kolin, KEB-EH030, Taipei, Taiwan) were used in shallow water where marine midges emerged and gathered. The volume was set at 80 dB for each loudspeaker. Five treatments of sound, at 75 Hz, 150 Hz, 225 Hz, 300 Hz, and a silent control, were used. The sound consisted of single tones for 8 s, followed by 10 s of silence. The cycle was then repeated; these were played for 3 min before the traps were retrieved and the number of midges was counted. Initially, the traps were placed at a distance of approximately 0.5 m from each other although local waves and wind tended to aggregate these traps. The test was repeated six times with different combinations of speakers and traps. A nonparametric Friedman test was used to compare the number attracted by different frequencies of sound in this experiment; then, the Wilcoxon signed rank test was used for pairwise comparisons.

In the second sound experiment, the background sound was masked by white noise at all frequencies (110 dB at 10-cm distance) by using the same speakers mentioned previously. We then tested whether midges were attracted to light from different directions. The experiment was performed in the 60-cm diameter basin described previously, with a no-white-noise group used as a control. The tests were alternating between the presence and absence of white noise so that both treatments could be tested over similar durations in the evenings. The counting and statistical methods followed that of the light experiment.

### Statistics

Circular statistics were used for experiments performed in the round basin. The evenness of the distribution of marine midges in the basin of individual tests was measured using the Rayleigh test, and the significance of the mean angles (directions) was tested using Von Mises distribution. Either aggregation or no aggregation was assigned to each test based on the P value (0.05 as the criterion) of the aforementioned statistics. The software Oriana v 4.0 was used to calculate parameters in each test. For an overall conclusion of whether aggregation of marine midges was dependent on treatments (e.g., sea direction and light direction), Fisher’s exact test was applied using individual tests as the unit of observation.

For the sound experiment of different frequencies, the nonparametric Friedman test was used to compare the five treatments of sound frequencies. The Wilcoxon signed rank test was then used to make pairwise comparisons. For the CO_2_ experiment, the Wilcoxon signed rank test was used to compare the number of marine midges moving in two opposite directions. All statistical calculations were performed using the software StatView.

## Results

### Light experiment

**Fig 4:**
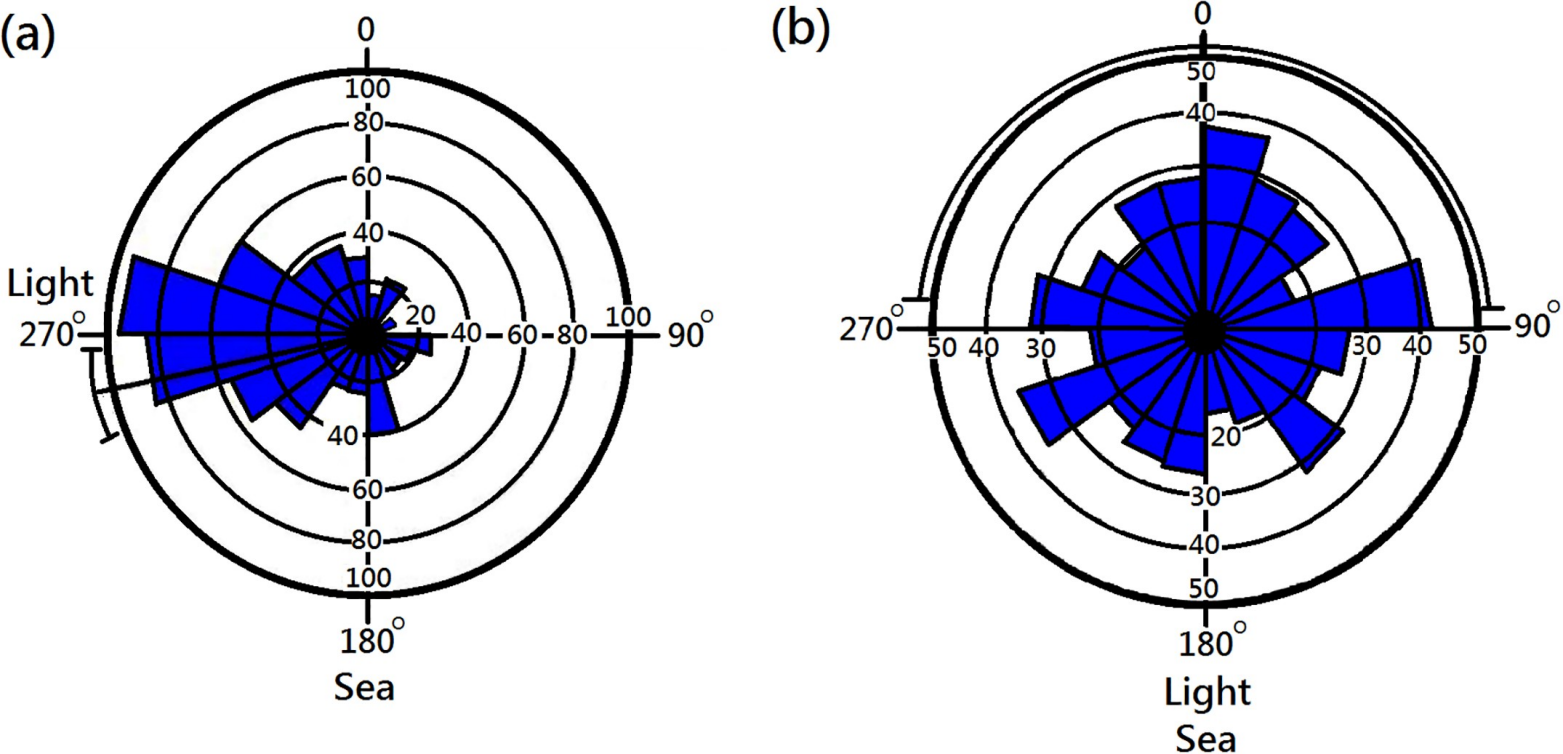
Typical distribution patterns of marine midges (*Pontomyia oceana*) in the experimental basin. (a) Light deployed away from the direction of the sea, significant aggregation (P < 0.05, Rayleigh test and Von Mises test), (b) Light deployed in the direction of the sea, no aggregation detected (P > 0.05, both tests).

Few tests could be performed within the duration when midges emerged each evening. Thus, the overall analysis was based on individual tests performed between November 16 and December 30, 2013. To increase sample sizes (i.e., number of basin-tests), data from the three locations, each with the sea facing a different direction, were pooled. We performed between 11 and 13 basin-tests in each location. Typically, midges gathered in the direction of the light although a few deviated from the light (Fig 4). For objectivity reason, aggregation or not was based on both the Rayleigh Test and the Von Mises Tests of the distribution patterns recorded on insect paper. For experiments with light spots deployed toward the sea, 1 of the 13 tests demonstrated significant aggregation. However, 14 of the 23 tests demonstrated significant aggregation in cases of light spots deployed away from the sea. Significant or insignificant aggregation in tests depended on the direction of the light spot (P < 0.01, Fisher’s exact test, Table 1). The light spot toward the sea was ineffective in inducing midge aggregation. None of the five total darkness tests demonstrated any significant aggregation.

**Table 1:** Analysis of independence between aggregation and light spot direction.

Significant Aggregation	Direction of light	
	Not sea	Sea
Yes	14	1
No	9	12

Fisher’s exact test, P < 0.01.

### Temperature experiment

None of the six tests, with three lower temperatures toward the sea and three toward the land, demonstrated any significant aggregation.

### CO_2_ experiment

**Fig 5:**
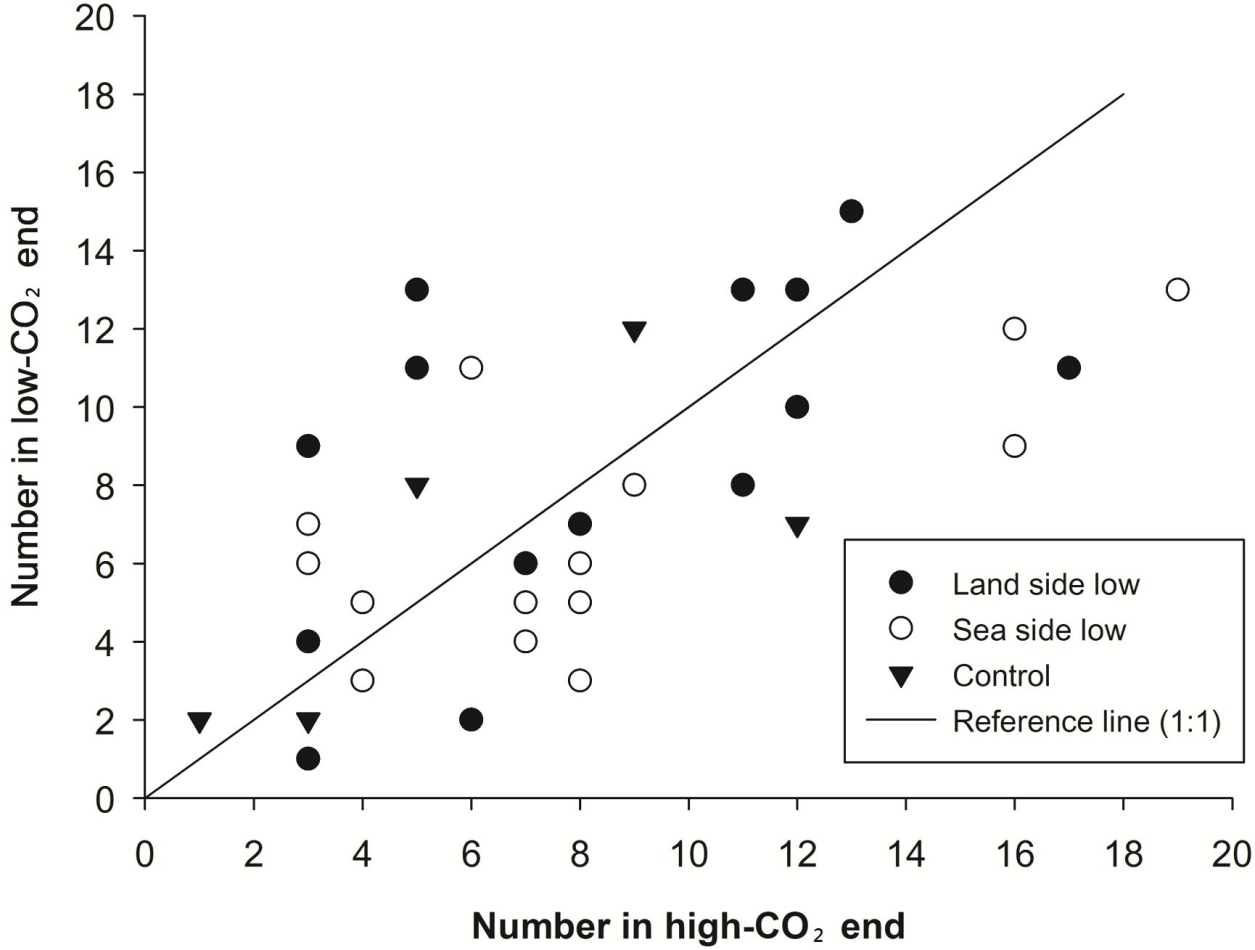
Distribution of midges (*Pontomyia oceana*) at the two ends in the CO_2_ gradient experiment. No difference was found between the two ends in any group. Treatments sharing the same alphabets have no significant differences among each other.

Average ambient pCO_2_ was 354 ppm on the evening of the experiment. The pCO_2_ level was low at the sea-facing end of the experimental tube at 159–194 ppm. Under this condition, more midges gathered at the end toward the sea than the opposite end in 9 of the 15 tests. When the low pCO_2_ end was facing the land, the pCO_2_ recorded was 167–185 ppm. In 8 of the 15 tests under this condition, more midges gathered at the end facing the land than the opposite end. No significant trend was observed in any of the above tests (sea lower: P = 0.31, land lower: P = 0.84, pooled: P = 0.55; Wilcoxon signed rank test) (Fig 5).

### Sound experiment

**Fig 6:**
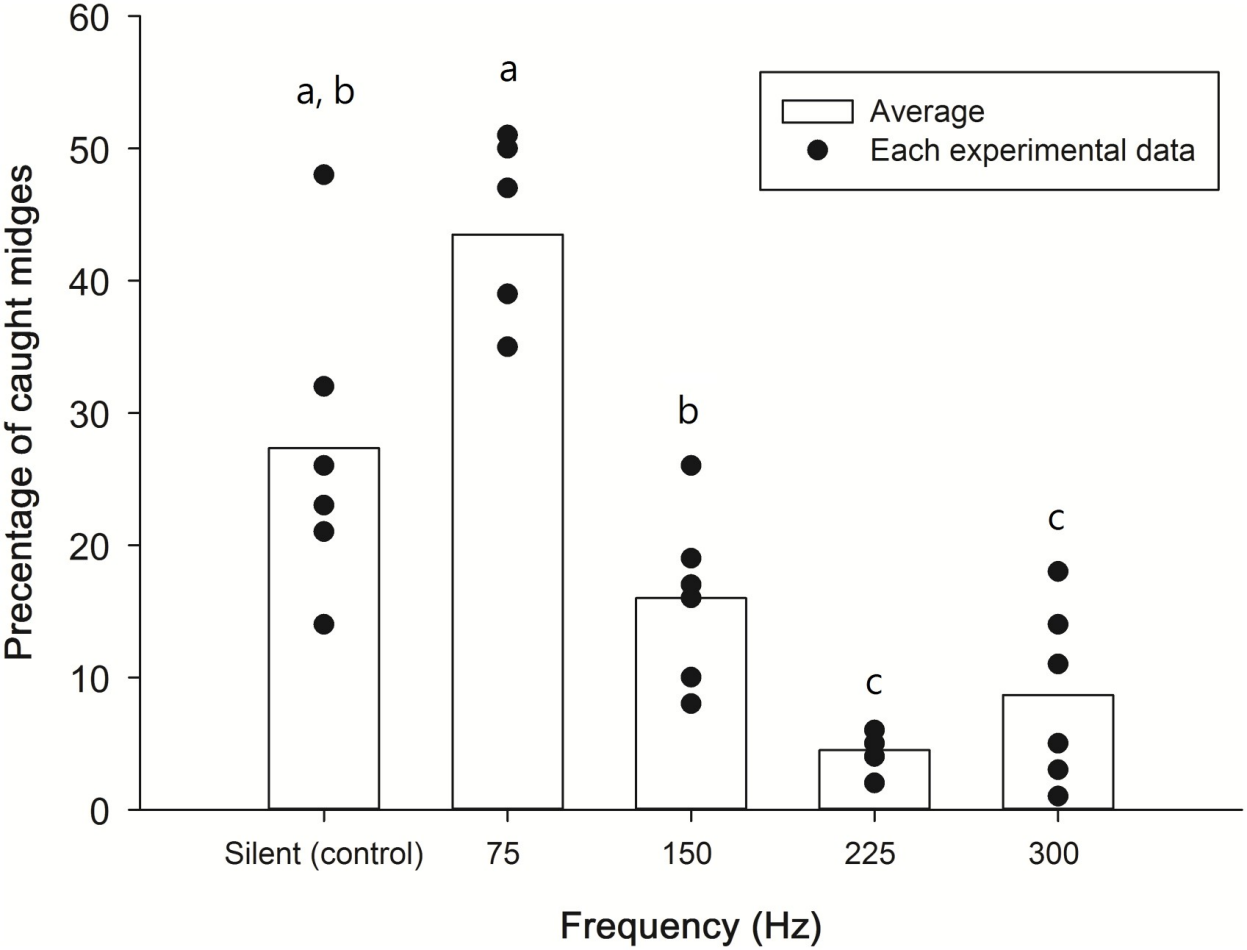
Percentage of marine midges (*Pontomyia oceana*) caught at different sound trap frequencies.

In the first part of five sound trap comparisons, the number of midges caught in each sound trap was compared. The 75-Hz trap had the highest number of midges caught in five of the six replicate tests, whereas traps with frequencies at 150 Hz, 225 Hz, and 300 Hz had lower numbers. The silent traps had intermediate numbers in most cases. The difference among treatments was significant (P < 0.01, Friedman test). Pairwise comparisons indicated that the 75 Hz versus silent (P = 0.06), silent versus 150 Hz (P = 0.17), and 225 Hz versus 300 Hz (P = 0.22) were not significantly different (Wilcoxon signed rank tests), whereas other combinations were (Fig 6).

In the white-noise masking experiment, 7 of the 12 tests with white noise treatment demonstrated significant aggregation with light spots facing the sea; by contrast, only 1 of the 10 tests demonstrated this phenomenon in the control test without white noise. The aggregation of midges with the light from the direction of the sea was dependent on the presence of white noise treatment (P = 0.03, Fisher’s exact test, Table 2).

**Table 2:** Test of independence between the aggregation and presence of white noise for *Pontomyia oceana*.

Significant aggregation	White noise	
	Yes	No
Yes	7	1
No	5	9

P = 0.03, Fisher’s exact test.

## Discussion

Our results clearly demonstrated that sound is used by the midge *P*. *oceana* to move toward light spots in the direction of the shore or rather to move away from the direction of the sea. This behavior could help the midge to locate hard substrates, which is more likely to occur in shallow waters toward the shore. In the evenings of midge emergence, this capability should be helpful, as several light reflection points or areas exist nearby, especially at moonlight. However, with reflection occurring both in the direction of the sea and the coast, relying solely on reflection is insufficient. The ability to use sound to detect the direction of the shore may thus evolve under such circumstances.

Regarding the question of whether background noise is helpful in the absence of moonlight, such as in the evenings around new moons, our results indicated that under total darkness, midges did not choose any particular direction and certainly not the direction of the coast. Thus, how they increase their chance of locating suitable substrates around new moons is unclear. In our experiments, we tested some likely environmental factors.

The experiment involving CO_2_ gradients was designed to examine one of these possibilities. In our experiments, the partial pressure of CO_2_ in the 4-m pipe had steep gradients; however, no evidence was found that midges used these gradients to orient themselves. In a preliminary pipe test, midges were observed to be sensitive to air composition, because extremely high pCO_2_ immobilized and rapidly killed midges. Mosquitoes are known to respond to a 150-ppm change in pCO_2_ to locate prey [23]. The marine midge *P*. *oceana* does not feed after it emerges as an adult. Thus, it does not have the motivation to search for food. With a speed of 30–80 cm/s, it took *P*. *oceana* 2.5–7 s to move 2 m on the water surface (personal observations of KS). This is well within the response time of the olfactory neurons in mosquitoes [24] if they indeed use CO_2_ gradients to guide their direction in locomotion. Its congeneric, *P*. *natans*, lay fertilized eggs on the water surface (personal observation of KS); hence, they do not require substrates for their eggs. Specifically, the search for a hard substrate may be a new trait in *P*. *oceana* as is the requirement to distinguish direction between the land and sea. Thus, *P*. *oceana* may had not enough evolutionary time to adapt to possible other available cues that optimize their targeting breeding habitats. Alternatively, this species is still in the evolutionary process to exploit possible environmental triggers of locomotory orientations and develop those optimally for their particular reproductive niche.

In the field, a CO_2_ gradient along the coast may not be homogeneous because of irregular coast lines and the distance between shallow habitats and the woods on land. pCO_2_ also fluctuates greatly on land between day and night, presumably because of the contrasting photosynthetic activity of land plants. In winter, when the diurnal emergence time is delayed until approximately 5 h after sunset [13], the CO_2_ gradient at the shore may demonstrate a reversed trend compared with that in the summer when emergence time is much sooner after dark. These factors may render the pCO_2_ gradient a less reliable predictor of where the land and shore are or rather where the exposed substrate is; thus, *P*. *oceana* is less likely to possess the ability to exploit this environmental cue.

Temperature differences between exposed hard substrates and seawater may be inconsistent. *Pontomyia oceana* in Taiwan reproduces all throughout the year; the diurnal emergence time is approximately 1 h after dusk in the summer but could be late [12]. The temperature of hard substrates above the sea surface must be higher than that of seawater during the day and early evening but may be lower than in water at late evenings since land has a much lower heat capacity than water. A marine midge may thus detect exposed substrates as spots of high temperature on some evenings and spots of low temperature at other times. This inconsistent difference may render temperature a useless factor for the marine midge to infer where the exposed substrate is.

Ambient sound, in comparison with the two previous factors, is not known to demonstrate a reversed trend between the sea and land directions in natural conditions within a few hours in one evening. It may thus be a more reliable indicator of where the coast is, especially in the darkness of the night.

What exactly produced the soundscape difference between the land and the sea is unclear, although waves breaking at the shore provide a reasonable and characteristic generator of coastal soundscapes. Our tests were performed at Wanliton, with a more-or-less protective reef structure, i.e., moving midges can easily encounter hard substrates in most directions. In other regions, the marine midge occurs at more open coasts, such as at Dongsha Island, the capability of finding shores and thus hard substrates to deposit eggs is critical.

Our experiments solved only half the challenge, that is, we determined how marine midges navigate in the evenings under the moonlight. However, how they locate suitable substrates in the absence of moonlight is still unclear.

*P*. *oceana* might start using wave-protected coasts, such as the one at Wanliton, initially. Once they have exploited a reliable environmental signal for their orientation, such as using sound to locate suitable habitats for egg deposition, their habitats could expand to cover new spaces including more open coasts, such as the ones at Dongsha Island. Marine insects may still have many other opportunities in the sea. However, they require an effective method of navigating in the direction of suitable substrates.

Our research also indicates that sound pollution may pose a threat to more inconspicuous marine inhabitants, whereas negative effects of sound pollution are better investigated in flagship species of marine conservation such as whales [25]. Since tiny marine midges use sound to locate the shore to lay eggs, they must also be sensitive to sound pollution. It is perceivable that up to 50% of adult marine midges could be lost due to polluting sound that masked the signal from normal shores. We recommend that policymakers in national parks should pay more attention to sound pollution issues, both from offshore and from land, such as during the tourist season.

## Supporting information

S1 Data (DOCX)

S2 Data (DOCX)

S3 Data (DOCX)

S4 Data (DOCX)
