# Supplementary material for: Navigation in darkness: How the marine midge (Pontomyia oceana) locates hard substrates above the water level to lay eggs
Source: PLoS One. 2021 Jan 25;16(1):e0246060. doi: 10.1371/journal.pone.0246060 (PMC7834138; doi:10.1371/journal.pone.0246060)
Supplement: S1 Data — (DOCX) [file pone.0246060.s001.docx]

**S1 Data. pCO_2_ in a transect on the reef flat perpendicular to the coast line at Wanliton.** A: sea end, B: land end.

| Distance from A to B | pCO_2_ (ppm) |
| --- | --- |
| 0 | 281 |
| 20 | 301 |
| 40 | 330 |
| 60 | 355 |
| 80 | 364 |
| 100 | 403 |
