# Supplementary material for: Navigation in darkness: How the marine midge (Pontomyia oceana) locates hard substrates above the water level to lay eggs
Source: PLoS One. 2021 Jan 25;16(1):e0246060. doi: 10.1371/journal.pone.0246060 (PMC7834138; doi:10.1371/journal.pone.0246060)
Supplement: S2 Data — (DOCX) [file pone.0246060.s002.docx]

**S2 Data. Difference in sound intensity detected by receivers pointing seaward vs landward at the shore of Wanliton, SW Taiwan.** Positive values indicate higher value facing the sea.

| Frequency (Hz) | Landward(dB) | Seaward (dB) |
| --- | --- | --- |
| 86.13281 | -22.3963 | -21.1446 |
| 172.2656 | -41.753 | -32.7078 |
| 258.3984 | -43.5976 | -44.9359 |
| 344.5313 | -44.6026 | -52.7965 |
| 430.6641 | -45.5866 | -59.0119 |
| 516.7969 | -47.2129 | -63.5629 |
| 602.9297 | -45.3265 | -67.4564 |
| 689.0625 | -43.4935 | -68.8321 |
| 775.1953 | -43.5909 | -69.5805 |
| 861.3281 | -45.0212 | -70.0622 |
| 947.4609 | -45.9246 | -70.5093 |
| 1033.594 | -45.1881 | -70.9227 |
| 1119.727 | -44.4532 | -70.7568 |
| 1205.859 | -44.225 | -70.9423 |
| 1291.992 | -44.4468 | -71.2157 |
| 1378.125 | -44.6598 | -71.4134 |
| 1464.258 | -44.1493 | -71.6229 |
| 1550.391 | -43.8339 | -72.2263 |
| 1636.523 | -44.1421 | -72.1346 |
| 1722.656 | -44.6284 | -72.0098 |
| 1808.789 | -45.5206 | -72.6717 |
| 1894.922 | -45.6531 | -73.5639 |
| 1981.055 | -45.4024 | -74.2516 |
| 2067.188 | -45.583 | -74.8984 |
| 2153.32 | -46.4732 | -75.421 |
| 2239.453 | -47.9287 | -75.9301 |
| 2325.586 | -48.0059 | -76.4207 |
| 2411.719 | -47.7825 | -77.4505 |
| 2497.852 | -47.929 | -78.3835 |
| 2583.984 | -48.9117 | -78.551 |
| 2670.117 | -49.0309 | -78.3715 |
| 2756.25 | -48.3934 | -78.3374 |
| 2842.383 | -47.556 | -78.832 |
| 2928.516 | -47.4199 | -77.6582 |
| 3014.648 | -47.8085 | -76.7511 |
| 3100.781 | -48.5092 | -78.1305 |
| 3186.914 | -47.8762 | -78.7737 |
| 3273.047 | -47.6651 | -79.2057 |
| 3359.18 | -48.5001 | -79.6769 |
| 3445.313 | -49.295 | -79.4495 |
| 3531.445 | -49.1226 | -78.9199 |
| 3617.578 | -48.314 | -78.8404 |
| 3703.711 | -48.2215 | -79.1606 |
| 3789.844 | -49.1281 | -79.404 |
| 3875.977 | -49.8207 | -79.6121 |
| 3962.109 | -49.981 | -79.3459 |
| 4048.242 | -49.6063 | -79.4784 |
| 4134.375 | -49.0822 | -79.6376 |
| 4220.508 | -49.2657 | -79.8304 |
| 4306.641 | -50.1842 | -79.9297 |
| 4392.773 | -50.3892 | -80.0807 |
| 4478.906 | -49.7291 | -80.1558 |
| 4565.039 | -49.5563 | -79.7794 |
| 4651.172 | -49.4472 | -80.0886 |
| 4737.305 | -49.7358 | -80.4522 |
| 4823.438 | -49.8014 | -81.1356 |
| 4909.57 | -49.839 | -81.5661 |
| 4995.703 | -50.4444 | -81.7746 |
| 5081.836 | -50.8577 | -82.0529 |
| 5167.969 | -50.9174 | -82.1463 |
| 5254.102 | -51.2422 | -82.5934 |
| 5340.234 | -51.4974 | -83.2176 |
| 5426.367 | -51.7816 | -83.6853 |
| 5512.5 | -52.1271 | -83.9744 |
| 5598.633 | -52.4687 | -84.4747 |
| 5684.766 | -52.8277 | -84.9206 |
| 5770.898 | -53.2918 | -85.8399 |
| 5857.031 | -53.6577 | -86.0082 |
| 5943.164 | -54.3595 | -85.7424 |
| 6029.297 | -55.0125 | -84.6827 |
| 6115.43 | -55.5849 | -85.7687 |
| 6201.563 | -56.108 | -87.5908 |
| 6287.695 | -57.0479 | -88.3901 |
| 6373.828 | -57.2647 | -88.8959 |
| 6459.961 | -57.5387 | -88.9523 |
| 6546.094 | -58.5467 | -88.8961 |
| 6632.227 | -59.1832 | -89.4293 |
| 6718.359 | -59.4598 | -90.4265 |
| 6804.492 | -59.3877 | -90.8329 |
| 6890.625 | -59.4959 | -91.1201 |
| 6976.758 | -59.8345 | -90.9854 |
| 7062.891 | -60.4094 | -91.6104 |
| 7149.023 | -60.6729 | -92.1634 |
| 7235.156 | -60.5799 | -92.2751 |
| 7321.289 | -60.6464 | -92.5179 |
| 7407.422 | -60.4235 | -92.5203 |
| 7493.555 | -60.5868 | -92.643 |
| 7579.688 | -60.8736 | -93.1884 |
| 7665.82 | -60.7948 | -93.8639 |
| 7751.953 | -60.7786 | -94.192 |
| 7838.086 | -61.1778 | -94.1247 |
| 7924.219 | -61.3914 | -94.0515 |
| 8010.352 | -61.3629 | -94.1301 |
| 8096.484 | -61.488 | -94.9175 |
| 8182.617 | -61.7676 | -95.3544 |
| 8268.75 | -61.7732 | -95.3381 |
| 8354.883 | -62.2957 | -95.4799 |
| 8441.016 | -62.5307 | -95.6898 |
| 8527.148 | -62.6217 | -95.8259 |
| 8613.281 | -63.0296 | -96.0117 |
| 8699.414 | -63.5533 | -96.1742 |
| 8785.547 | -64.1123 | -96.2177 |
| 8871.68 | -64.6285 | -96.1148 |
| 8957.813 | -64.9528 | -96.0647 |
| 9043.945 | -65.0877 | -95.9432 |
| 9130.078 | -65.2412 | -96.0269 |
| 9216.211 | -65.6871 | -96.3829 |
| 9302.344 | -66.4735 | -96.4645 |
| 9388.477 | -66.7552 | -96.4043 |
| 9474.609 | -66.6107 | -96.5214 |
| 9560.742 | -66.7438 | -96.7327 |
| 9646.875 | -67.6907 | -96.8127 |
| 9733.008 | -68.2256 | -97.3552 |
| 9819.141 | -68.218 | -97.4607 |
| 9905.273 | -67.827 | -97.5702 |
| 9991.406 | -67.8667 | -97.8909 |
| 10077.54 | -68.8922 | -98.2807 |
| 10163.67 | -69.8182 | -98.5006 |
| 10249.8 | -70.0404 | -98.4669 |
| 10335.94 | -69.7392 | -98.543 |
| 10422.07 | -69.4757 | -98.7924 |
| 10508.2 | -70.4063 | -99.2334 |
| 10594.34 | -71.3592 | -99.5959 |
| 10680.47 | -72.0086 | -99.7009 |
| 10766.6 | -71.6533 | -100.025 |
| 10852.73 | -71.6545 | -100.225 |
| 10938.87 | -72.9901 | -100.567 |
| 11025 | -73.8641 | -100.654 |
| 11111.13 | -74.219 | -100.629 |
| 11197.27 | -73.816 | -100.539 |
| 11283.4 | -73.9147 | -100.912 |
| 11369.53 | -75.2386 | -101.474 |
| 11455.66 | -75.8423 | -101.238 |
| 11541.8 | -75.9882 | -101.316 |
| 11627.93 | -75.0529 | -101.466 |
| 11714.06 | -75.0039 | -101.656 |
| 11800.2 | -76.2964 | -101.926 |
| 11886.33 | -76.4372 | -101.87 |
| 11972.46 | -76.5086 | -101.932 |
| 12058.59 | -75.379 | -101.761 |
| 12144.73 | -75.2123 | -101.576 |
| 12230.86 | -76.1297 | -101.734 |
| 12316.99 | -76.2746 | -101.971 |
| 12403.13 | -76.2706 | -102.009 |
| 12489.26 | -75.1292 | -101.707 |
| 12575.39 | -75.076 | -101.965 |
| 12661.52 | -75.9582 | -102.07 |
| 12747.66 | -76.2694 | -102.025 |
| 12833.79 | -76.1431 | -102.105 |
| 12919.92 | -75.082 | -102.133 |
| 13006.05 | -75.7005 | -101.958 |
| 13092.19 | -76.4343 | -101.767 |
| 13178.32 | -76.541 | -102.031 |
| 13264.45 | -76.297 | -101.894 |
| 13350.59 | -74.9286 | -101.866 |
| 13436.72 | -75.4374 | -101.738 |
| 13522.85 | -76.2294 | -101.709 |
| 13608.98 | -76.2303 | -101.72 |
| 13695.12 | -76.1081 | -101.979 |
| 13781.25 | -75.0639 | -101.988 |
| 13867.38 | -75.8454 | -101.988 |
| 13953.52 | -76.5099 | -101.609 |
| 14039.65 | -76.2505 | -101.356 |
| 14125.78 | -76.1657 | -101.844 |
| 14211.91 | -75.3981 | -101.796 |
| 14298.05 | -76.19 | -101.797 |
| 14384.18 | -76.4931 | -101.943 |
| 14470.31 | -76.7316 | -102.052 |
| 14556.45 | -76.6899 | -101.825 |
| 14642.58 | -75.6864 | -101.757 |
| 14728.71 | -76.7645 | -102.081 |
| 14814.84 | -77.1176 | -101.879 |
| 14900.98 | -77.1002 | -101.86 |
| 14987.11 | -76.8599 | -102.083 |
| 15073.24 | -76.0643 | -102.01 |
| 15159.38 | -76.9598 | -101.951 |
| 15245.51 | -77.3046 | -102.176 |
| 15331.64 | -77.5346 | -102.221 |
| 15417.77 | -77.105 | -101.65 |
| 15503.91 | -76.1841 | -101.535 |
| 15590.04 | -77.3321 | -101.863 |
| 15676.17 | -77.8185 | -101.772 |
| 15762.3 | -77.6744 | -101.968 |
| 15848.44 | -76.9371 | -102.115 |
| 15934.57 | -76.1772 | -102.073 |
| 16020.7 | -77.6189 | -102.178 |
| 16106.84 | -77.7542 | -102.294 |
| 16192.97 | -77.7424 | -101.97 |
| 16279.1 | -77.2846 | -101.78 |
| 16365.23 | -76.8542 | -102.029 |
| 16451.37 | -78.0417 | -102.328 |
| 16537.5 | -77.9829 | -102.359 |
| 16623.63 | -78.3411 | -102.138 |
| 16709.77 | -77.4896 | -101.827 |
| 16795.9 | -76.9366 | -101.71 |
| 16882.03 | -78.1164 | -101.918 |
| 16968.16 | -78.4191 | -101.925 |
| 17054.3 | -78.523 | -101.948 |
| 17140.43 | -77.6288 | -102.056 |
| 17226.56 | -77.1088 | -102.079 |
| 17312.7 | -78.2637 | -102.124 |
| 17398.83 | -78.4746 | -102.156 |
| 17484.96 | -78.2996 | -102.13 |
| 17571.09 | -77.3209 | -101.987 |
| 17657.23 | -77.3982 | -102.268 |
| 17743.36 | -78.4535 | -102.078 |
| 17829.49 | -78.6333 | -101.955 |
| 17915.63 | -78.6451 | -102.122 |
| 18001.76 | -77.6116 | -102.011 |
| 18087.89 | -77.9242 | -101.981 |
| 18174.02 | -78.7899 | -102.225 |
| 18260.16 | -78.8123 | -102.214 |
| 18346.29 | -78.8179 | -102.201 |
| 18432.42 | -77.7063 | -102.241 |
| 18518.55 | -78.0576 | -102.21 |
| 18604.69 | -79.1047 | -101.879 |
| 18690.82 | -79.3172 | -101.682 |
| 18776.95 | -78.8291 | -102.073 |
| 18863.09 | -77.6498 | -102.041 |
| 18949.22 | -78.1163 | -102.15 |
| 19035.35 | -78.8634 | -102.264 |
| 19121.48 | -79.1093 | -102.189 |
| 19207.62 | -79.0348 | -102.203 |
| 19293.75 | -77.8546 | -102.037 |
| 19379.88 | -78.6183 | -102.31 |
| 19466.02 | -79.4439 | -102.237 |
| 19552.15 | -79.33 | -102.022 |
| 19638.28 | -79.1838 | -102.156 |
| 19724.41 | -78.2262 | -102.23 |
| 19810.55 | -78.8458 | -102.37 |
| 19896.68 | -79.6058 | -102.226 |
| 19982.81 | -79.6049 | -102.126 |
| 20068.95 | -79.095 | -102.111 |
| 20155.08 | -78.3203 | -102.075 |
| 20241.21 | -79.2513 | -102.338 |
| 20327.34 | -79.8205 | -102.328 |
| 20413.48 | -79.6963 | -102.298 |
| 20499.61 | -79.2218 | -102.213 |
| 20585.74 | -78.6273 | -102.045 |
| 20671.88 | -79.4782 | -102.324 |
| 20758.01 | -79.8007 | -102.49 |
| 20844.14 | -79.8276 | -102.491 |
| 20930.27 | -79.4768 | -102.147 |
| 21016.41 | -79.0272 | -102.139 |
| 21102.54 | -80.1063 | -102.614 |
| 21188.67 | -80.1066 | -102.676 |
| 21274.8 | -80.0764 | -102.379 |
| 21360.94 | -79.7533 | -102.326 |
| 21447.07 | -79.4 | -102.542 |
| 21533.2 | -80.4099 | -102.651 |
| 21619.34 | -80.6731 | -102.566 |
| 21705.47 | -80.3294 | -102.445 |
| 21791.6 | -79.5901 | -102.178 |
| 21877.73 | -79.5167 | -102.411 |
| 21963.87 | -80.309 | -102.574 |
