# Supplementary material for: Navigation in darkness: How the marine midge (Pontomyia oceana) locates hard substrates above the water level to lay eggs
Source: PLoS One. 2021 Jan 25;16(1):e0246060. doi: 10.1371/journal.pone.0246060 (PMC7834138; doi:10.1371/journal.pone.0246060)
Supplement: S3 Data — (DOCX) [file pone.0246060.s003.docx]

| **Control** | | **Land side low** | | **Sea side low** | |
| --- | --- | --- | --- | --- | --- |
| **Number in high CO_2_** | **Number in low CO_2_** | **Number in high CO_2_** | **Number in low CO_2_** | **Number in high CO_2_** | **Number in low CO_2_** |
| 1 | 2 | 3.0000 | 1.0000 | 3.0000 | 6.0000 |
| 3 | 2 | 3.0000 | 4.0000 | 3.0000 | 7.0000 |
| 5 | 8 | 3.0000 | 9.0000 | 4.0000 | 3.0000 |
| 12 | 7 | 6.0000 | 2.0000 | 4.0000 | 5.0000 |
| 9 | 12 | 5.0000 | 11.0000 | 6.0000 | 11.0000 |
|  |  | 5.0000 | 13.0000 | 7.0000 | 4.0000 |
|  |  | 7.0000 | 6.0000 | 7.0000 | 5.0000 |
|  |  | 8.0000 | 7.0000 | 8.0000 | 3.0000 |
|  |  | 11.0000 | 8.0000 | 8.0000 | 5.0000 |
|  |  | 11.0000 | 13.0000 | 8.0000 | 6.0000 |
|  |  | 12.0000 | 10.0000 | 9.0000 | 8.0000 |
|  |  | 12.0000 | 13.0000 | 16.0000 | 9.0000 |
|  |  | 13.0000 | 15.0000 | 16.0000 | 12.0000 |
|  |  | 17.0000 | 11.0000 | 19.0000 | 13.0000 |

**S3 Data. Distribution of midges (*Pontomyia oceana*) at the two ends in the CO_2_-gradient experiment.** No difference was found between the two ends in any group.
