# Supplementary material for: Navigation in darkness: How the marine midge (Pontomyia oceana) locates hard substrates above the water level to lay eggs
Source: PLoS One. 2021 Jan 25;16(1):e0246060. doi: 10.1371/journal.pone.0246060 (PMC7834138; doi:10.1371/journal.pone.0246060)
Supplement: S4 Data — (DOCX) [file pone.0246060.s004.docx]

**S4 Data. Percentage of the marine midges ((*Pontomyia oceana*) caught in different frequency of sound trap.**

| control | 75Hz | 150Hz | 225Hz | 300Hz |
| --- | --- | --- | --- | --- |
| 21% | 50% | 19% | 5% | 5% |
| 48% | 35% | 8% | 6% | 3% |
| 32% | 51% | 10% | 5% | 1% |
| 14% | 47% | 26% | 2% | 11% |
| 23% | 39% | 16% | 4% | 18% |
| 26% | 39% | 17% | 5% | 14% |
